# Effect of climate and geography on worldwide fine resolution economic activity

**DOI:** 10.1371/journal.pone.0229243

**Published:** 2020-03-02

**Authors:** Alberto Troccoli

**Affiliations:** World Energy and Meteorology Council, The Enterprise Centre, University of East Anglia, Norwich, England, United Kingdom; Xiamen University, CHINA

## Abstract

Geography, including climatic factors, have long been considered potentially important elements in shaping socio-economic activities, alongside other determinants, such as institutions. Here we demonstrate that geography and climate variables satisfactorily explain the worldwide economic activity as measured by the per capita Gross Cell Product (GCP-PC) at a fine geographical resolution, typically much higher than country average. A 1° by 1° GCP-PC dataset has been key for establishing and testing a direct relationship between ‘local’ geography/climate and GCP-PC. Not only have we tested the geography and climate hypothesis using many possible explanatory variables, importantly we have also predicted and reconstructed GCP-PC worldwide by retaining the most significant predictors. While this study confirms that latitude is the most important predictor for GCP-PC when taken in isolation, the accuracy of the GCP-PC prediction is greatly improved when other factors mainly related to variations in climatic variables, rather than average climatic conditions as typically used, are considered. However, latitude diminishes in importance when only the wealthier parts of the globe are considered. This work points to specific features of the climate system which explain economic activity, such as the variability in air pressure. Implications of these findings range from an improved understanding of why socio-economically better-off societies are geographically placed where they are in the present, past and future to informing where new economic activities could be established in order to yield favourable economic outcomes based on geography and climate conditions.

## Introduction

The relevance of meteorology (comprising weather and climate) and geography variables on the socio-economic activities is a fascinating subject and one that has been touched on intermittently for centuries. Notable mentions are Montesquieu’s treatise ‘The Spirit of Laws’ of 1750 in which, as reported by [1], he argued that an ‘excess of heat’ made men ‘slothful and dispirited’. More than a century and a half later, [2] analysed in more detail how key weather variables such as temperature and humidity influence labour productivity. He found that not only did temperature have an effect on labour productivity, also other meteorological factors such as relative humidity, wind speed, and storms (or pressure changes) were important too. He also considered large scale patterns and speculated that changes in the global civilisation might be linked to centennial variations in climate. It is this macro-scale approach, rather than the more specific but motivational labour productivity work of [2], that is the focus of our work.

There is no doubt that economic development is a highly complex phenomenon, one which inextricably links physical and social factors. It is nonetheless possible, and highly interesting, to investigate how individual determinants contribute to it [e.g. 3, 4]. While full consideration has been given to representing a wide range of determinants, this work focuses on the role of geography and climate for reasons explained below.

Only sporadic studies followed the work of Huntington, until the last few decades when [3, 5–7] re-elaborated and advanced our understanding about the importance of geography and climate in economic-growth studies. Possibly the main reason for the sporadic studies in the twentieth century is that these *became associated with racism because it intimated that people in the tropics were less productive than those in the temperate zone* [8]. However, the use of improved data and methodologies in the last few decades have led to much more solid research results and therefore rendered that criticism seemingly anachronistic. Investigations that have taken into account geography and climate to explain socio-economic activity normally use an invariant geography variable, latitude, and just a few climatic variables, mainly mean temperature and precipitation, as explanatory factors [1, 4, 9]. The corollary of this is that latitude, mean temperature and precipitation (individually or in combination) essentially have become synonym of geography and climate in a large component of the current literature in this area. This has crucial implications because, albeit important, mean temperature and precipitation are just two of the variables regulating weather and climate. Therefore there is a risk to dismiss the geography and climate argument just because it is not always possible to explain economic activity by just using these variables. That is why it is critical to consider meteorology, together with geography, in a more holistic way, as we do in this work.

One of the limited examples where more geography and climate variables are considered is for the case of African economic activity [10]. By using linear and squared terms in mean precipitation, mean temperature, elevation, and the distance from coastline, lakes, and rivers, [10] concluded that these explain a substantial proportion of the economic output for Africa. In [11] determinants of economic development covering 1867 subnational regions from 101 countries, focusing on *within*-country effects of geography and institutions using 25 geography and mean climate variables were investigated. It was concluded that while institutions have a significant positive effect on income among subnational regions with greater autonomy, [11] found that, *simply put*, *geography matters*.

The question this work addresses is: “*What is the role of climate and geography in worldwide fine resolution economic activity and specifically which climatic/geographical variables are the most relevant for economic activity*?” To achieve this, we considerably extend the number, and crucially the type, of geography and climate variables used by [10, 11]. More specifically, in addition to the mean of variables such as temperature, precipitation, air pressure, relative humidity, dew point temperature, wind speed, solar radiation and sunshine duration, we consider their variations in time. The idea behind the use of variations in climate variables is that we can for instance mimic the effect of decreasing air pressure, which is akin to an incoming weather perturbation, such as a storm. This approach allows us to assess the effect of wider meteorological variables on the worldwide economic activity, as measured by the per capita Gross Cell Product (GCP). The conceptual basis of GCP is the same as that of gross domestic product (GDP) and gross regional product as developed in the national income and product accounts of major countries, except that the geographic unit is the latitude-longitude grid cell [10].

This work contributes to the debate about the role geography and climate have in shaping communities and economies. Such a debate has been driven by two fundamental determinants: institutions versus geography and climate (some authors include also a third determinant, international trade or market integration [12, 13], which is however itself dependent on institutions, and geography). On the one hand, the popular book [14], and before it [4, 15, 16], have been arguing about the criticality of the role of institutions in driving productivity. [4] attempted a quantification of the respective roles of institutions, geography/climate (the latter simulated by latitude only), but also integration, and concluded that *once institutions are controlled for*, *integration has no direct effect on incomes*, *while geography has at best weak direct effects*.

However, because institutions are the result of a wider variety of factors, both exogenous and endogenous, including human, geography and climate factors, it is more difficult to disentangle causes and effects. For instance, as expressed in [17], one of the reasons why European settlers did not establish themselves in tropical areas (e.g. sub-Saharan Africa) is the presence of diseases such as yellow fever and malaria which are prevalent in tropical climatic conditions.

On the other hand, it is difficult to identify appropriate institution-related indicators that are truly statistically independent of GDP. Of the six indicators considered by [18]–*Voice and Accountability*, *Political Stability and Absence of Viole*nce, *Government Effectiveness*, *Regulatory Quality*, *Rule of Law*, and *Control of Corruption*–their individual linear correlation coefficient with GDP ranges between 0.6 and 0.9, and predominantly at the higher end of this range. For instance, *Rule of law*, as used in [4], has a coefficient of ca. 0.8 with GDP. More importantly, however, a key limitation with institution indicators is that they are not available globally at a resolution finer than country averages, which is essential for the work performed here.

The most attractive feature of trying to explain economic development through geography and climate factors is that these are essentially exogenous elements. In fact, *because weather is exogenous and random in most economic applications*, *it acts like a “natural experiment”* thus allowing the identification of relationships between economic outcomes and meteorology in a scientific way [19, 20].

In this study we take advantage of a high-resolution global GCP data set [21]. The lack of a granular GCP global map has prevented researchers from intimately assessing the connection between geography/climate and economic activity. And although many attempts have been made [7 and references therein], studying their link at the level of country-average, i.e. using Gross Domestic Product (GDP), is sub-optimal. In fact, climate variables can vary wildly within a country. This is certainly true for very large countries like USA, China or Australia, but even for smaller countries like Italy, with a distinct north-south gradient in both climate and productivity, relying on GDP is not satisfactory [22].

Another important extension introduced here is the use of a non-linear model. Most of the current literature simulates GCP variations using (multi)linear models, including [11]. Although practical and reasonable, linear models have major limitations, particularly in a complex non-linear problem like the modelling of economic activity.

This study shows that climate and geography variables accurately explain, and reproduce, the per capita GCP worldwide at a fine resolution. In addition to latitude, less obvious variables such as month-to-month changes in air pressure are the next most important climate predictors. Our results provide a non-linear machine-learning framework for assessing and quantifying the main climate and geography factors affecting economic activity. These results, purely based on exogenous variables, have implications for understanding what could be favourable environments for thriving economies, or otherwise.

The next section, 2, presents the data sets used, while the methodology is discussed in Section 3 and results are examined in Section 4. A summary and discussion are provided in Section 5.

## Data

The underlying meteorological data are provided by the ERA-Interim reanalysis. This reanalysis is produced by the European Centre for Medium-Range Weather Forecasts (ECMWF) and is described in [23]. Here we use data from 1979 to 2016, i.e. most of the ERA-Interim available period. Its main features are a horizontal resolution of 0.75° by 0.75°, and a temporal resolution which varies between 3 hours and 6 hours, depending on the variable (see Table 1). Also, several derivatives of the meteorological variables considered, and listed in Table 1, are used.

**Table 1:** Meteorological variables used in this study, as derived from the ERA-Interim reanalysis. The following statistics have been computed for all variables: Mean, 1^st^ Quartile (bottom Q), Median, 3^rd^ Quartile (top Q), Standard Deviation of the original time series (SD), Standard Deviation of monthly means (SD S, representing intra-annual variations) and temporal variations (representing short-term ‘gradients’). The latter are computed according to daily or 6-hourly steps (see column Step). For 6-hourly variables, increments are increased by 10% at each subsequent steps (out to 5 steps, i.e. 30 hours) and by 15% for daily steps (out to 5 days). For air temperature (at 2 m height), also daily excursions are calculated using two additional variables, T_min_ and T_max_, available at 6-hour intervals.

Climate Variable	Unit	‘gradient’	Step
Mean Sea Level Pressure (MSLP)	hPa	750	6-hr
Wind Speed at 10m (UV10)	m s^-1^	2.5	6-hr
Air Temperature (T2)	°C	5	Daily
Air Temperature daily excursion (DT)	°C	2.5	Daily
Dew Point Temperature (D2)	°C	7	Daily
Precipitation (TP)	mm	5	Daily
Relative Humidity (RH)	%	20	Daily
Solar Radiation (SR)	W m^-2^	100	Daily
Sunshine Duration (SUND)	hr	3	Daily

The globally gridded GCP data used here comes from the Global Gridded Geographically Based Economic Data (G-Econ, https://gecon.yale.edu/data-and-documentation-g-econ-project), Version 4 [21] (see also an early version, for 1990 only, in [6]). This dataset contains derived one-degree grid cells of GCP data for the years 1990, 1995, 2000 and 2005. To allow for an easier direct comparison across the globe, we consider GCP at Purchasing Power Parity and per capita. Per capita GCP (hereafter referred to as GCP-PC) is computed by dividing GCP by the population in each cell, also provided with the G-Econ dataset.

While the main use of the G-Econ is the gridded GCP data, this dataset also includes geographical parameters such as area of grid cells, distance to coast, elevation, vegetation and soil types, distance to rivers, which are also used in our analysis, along with the climate variables (see Table 2). All geography parameters are fixed for the period covered by this study–possible local variations particularly in vegetation or soil types, e.g. around urban centres, do not appear to be critical and they vary on a timescale slower than those of climate variables. To ensure geographical overlap with G-Econ, ERA-Interim is retrieved at 1°, namely at a slightly lower resolution than its original 0.75°.

**Table 2:** Geography parameters. Aside from latitude and elevation (the latter has been taken from the ERA-Interim reanalysis), the other variables are from the G-Econ dataset.

Geography parameter	Unit
Latitude	Degrees
Elevation	m
Distance to coast 1	km
Distance to coast 2	km
Distance to Lake	km
Distance to Major River	km
Distance to River	km
Distance to Ocean	km
Vegetation category	category [0–31]
Soil category	category [0–250]

## Assumptions and methodology

The main focus of this work is the investigation of the casual relationship between the main features of meteorological variables over few decades and the corresponding GCP, rather than the (concurrent) correlation between meteorological variables and GCP. These two objectives require different approaches. Specifically, in the first case statistical properties such as seasonal variations of meteorological variables are used. It is this type of features, namely the changes in variables, that we want to analyse in addition to the more standard statistics such as the mean (of e.g. temperature). Accordingly, the main assumption here is that the statistics of both the meteorology and GCP are stationary over the period considered, namely 1979–2016 for the meteorological variables, and the four years of G-Econ, 1990, 1995, 2000 and 2005 for the GCP. A temporal correlation between GCP and climate, while possible, would not yield robust statistical results as GCP is only available for four years. Clearly within any given period the climate will vary, but the wide-ranging statistical characteristics we use here are designed to take into accounts such variations. More importantly, GCP changes in parts of the globe, particularly for China, over the G-Econ period. Accordingly, the stationarity assumption for GCP has been tested by perturbing it with GCP plus and minus one standard deviation (computed using the four years available), respectively: in both cases, the main predictors are the same as for the (mean) GCP, thus confirming the validity of this assumption (not shown).

**Fig 1:**
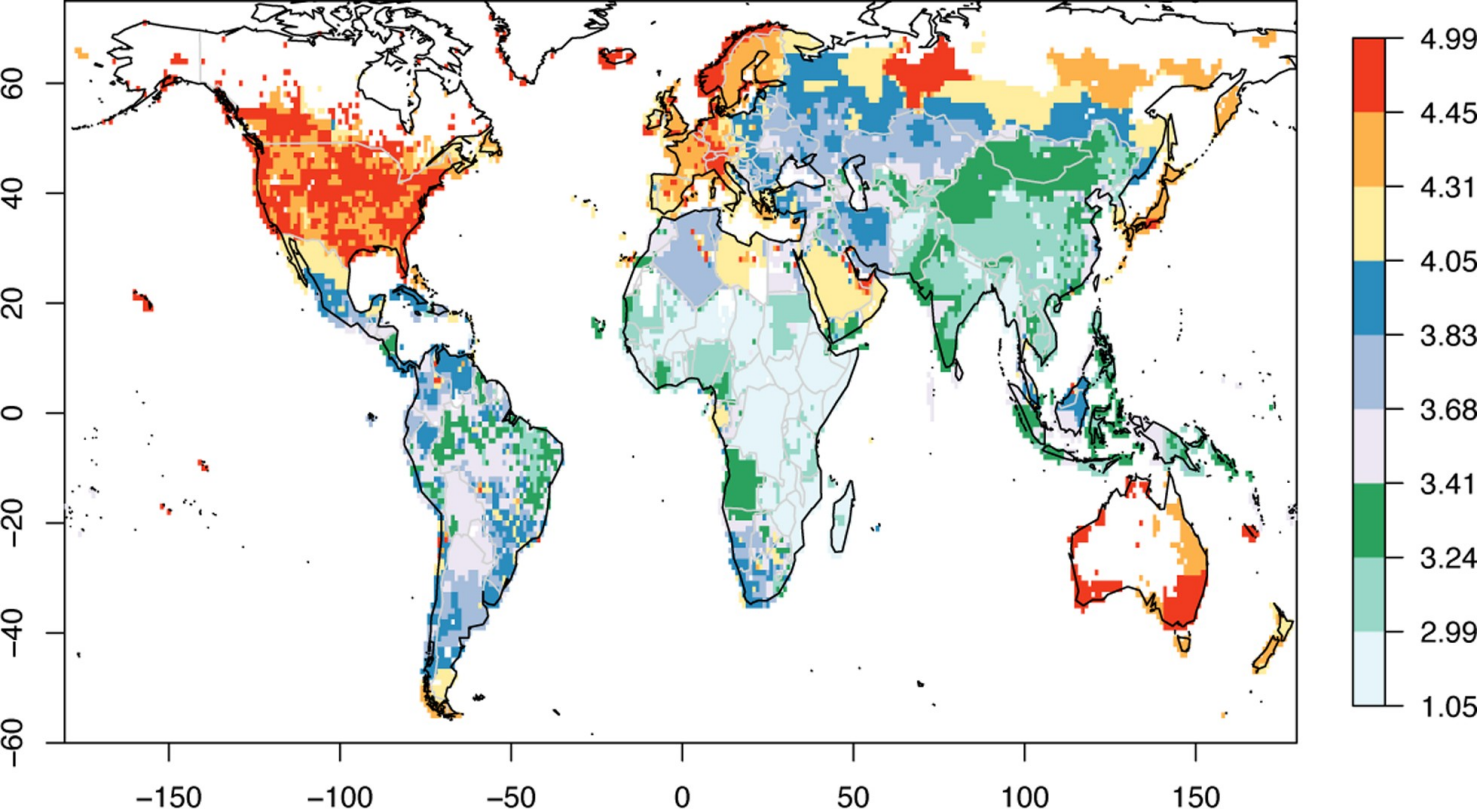
Map of log Gross Cell Product per capita. GCP per capita in log_10_ (k USD/person). Note that the colour palette has been divided into three sets of three colours each, which represent the three terciles, whose thresholds are 3.41 and 4.05, respectively (cf. Fig 2). Source G-Econ [21].

**Fig 2:**
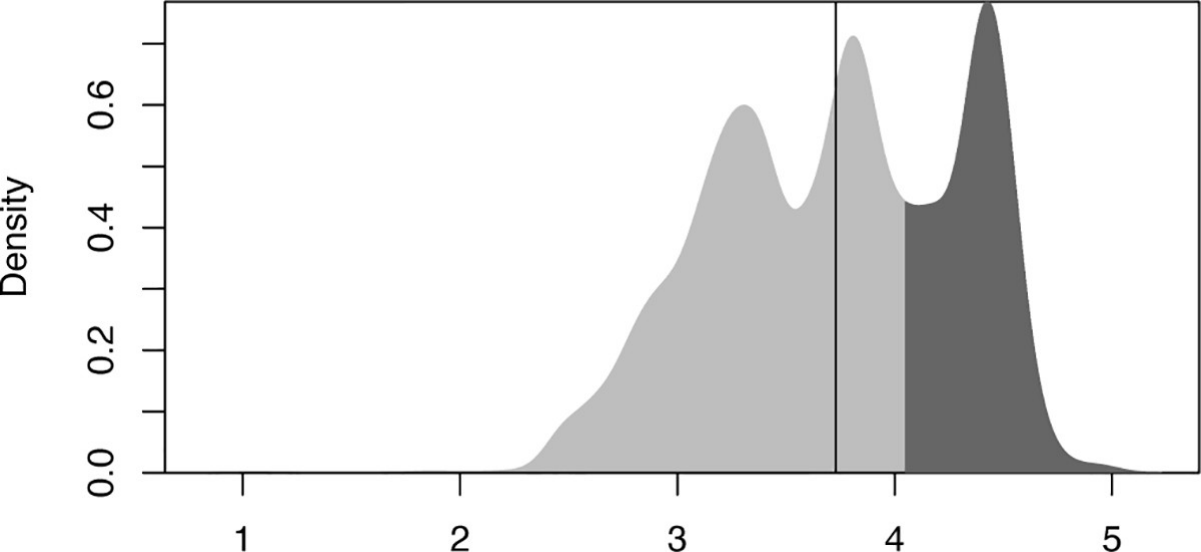
PDF of Gross Cell Product per capita. Probability Density Function (PDF) for log_10_ of GCP-PC. The black vertical line shows the mean of the distribution which is 3.73 (equivalent to about $5350/person). The PDF displays an interesting tri-modal behaviour (cf. also Fig 1): the first mode (between 3.05 and 3.55) corresponds to most of East and South Asia, Central-Northern Brazil, and parts of Western Africa; the second mode (between 3.55 and 4.1) covers Eastern Europe, Northern and Southern Africa, most of Central America and the rest of South America; the third mode (between 4.1 and 4.65) covers North America, Western Europe, parts of Russia, the Arabian peninsula, Japan and coastal Australia. The darker grey denotes the upper tercile.

Because a single target (or predictand), namely the GCP for each geographical location, is required, this is taken as the average of the four years in G-Econ. To reduce the noise in the analysis, grid cells where the GCP is smaller than 1 USD are not considered. Also, as normally done [e.g. 4], GCP is converted into a logarithm (base 10). The (log of the) GCP thus computed is shown in S1 Fig while the corresponding GCP-PC can be seen in Fig 1. Its Probability Density Function (PDF) is shown in Fig 2.

As already remarked, the focus of this work is the assessment of the dependency of GCP-PC on climate and geography variables. Formally this can be expressed as:

$$\begin{array}{c} \mathrm{GCP}‐\mathrm{PC}=f(\mathrm{Geography},\;\mathrm{Climate})+\underset{︸}{h(\mathrm{Institutions},\;\mathrm{National}\;\mathrm{Resources},\ldots )+\varepsilon }\\ \mathrm{GCP}‐\mathrm{PC}=f(\mathrm{Geography},\;\mathrm{Climate})+\delta \end{array}$$ (1)

where *ε* is the error term, which captures factors controlling GCP-PC not accounted for by the other terms, therefore including errors in GCP, climate and geography variables; *δ* is a term not explicitly modelled in this work. By assessing the spatial geographical patterns of *δ* it may be possible to identify the source of potential mismatches, or residual, between GCP-PC and the geography and climate predictors used here through the modelled function, *f*. However, a direct assessment of the role of institutions is discussed in the next section.

The main tool used in our analysis is a non-linear statistical model called Random Forests (RF). This is a well-known and popular method consisting of a set of decision trees built to minimise their correlation [24, 25]. The model has been chosen after a comparison with other models, Gradient Boosting (GB, a non-linear statistical model) [25] and the multi-linear (ML) regression model, following extensive tests of tuning hyper-parameters, using the *caret* (Classification And REgression Training) package in R. With the RF model the issue of overfitting due to collinearity of variables (or predictors) is considerably reduced, or even eliminated [24]. The justification for using a non-linear model stems from the complex relationships between any one variable and GCP PC. These have been assessed through scatter plots, also reflected in the low correlation coefficients (see later Table 4) as well as, critically, by the low performance of the multi-model approach (see Table 3).

**Table 3:** Comparison statistics for different number of variables and different models (RF, GB and ML) and for all grid points (first two columns), the top tercile of GCP-PC (third and fourth columns, the middle tercile of GCP-PC (fifth and sixth columns), the bottom tercile of GCP-PC (last two columns). OOB stands for Out-of-bag prediction (a feature of the RF model). The top 10 predictors have been selected using the procedure described in the Methodology section. The row with the top 10 predictors for RF, which is used in the rest of the study, has been highlighted in grey.

	All grid points		Top tercile		Middle tercile		Bottom tercile	
	nMAE	CORR	nMAE	CORR	nMAE	CORR	nMAE	CORR
RF 121 (all) variables (OOB)	0.152	0.970	0.276	0.893	0.386	0.840	0.251	0.909
RF 121 (all) variables (5-fold)	0.162	0.966	0.291	0.885	0.403	0.830	0.262	0.906
GB 121 (all) variables (5-fold)	0.346	0.874	0.449	0.755	0.526	0.699	0.386	0.822
RF top 10 predictors (5-fold)	0.153	0.970	0.262	0.898	0.385	0.839	0.250	0.910
GB top 10 predictors (5-fold)	0.368	0.862	0.500	0.695	0.534	0.690	0.417	0.800
ML top 10 predictors (5-fold)	0.600	0.679	0.764	0.314	0.730	0.453	0.661	0.533

We use around 120 geography and climate predictors (see Tables 1 and 2). By construction many of these predictors are highly correlated. However, since the RF model can deal very well with collinearities [24], correlated variables are not eliminated. Instead, by retaining them we allow for multiple (correlated) predictors to emerge when assessing the variable ranking. If two or more highly correlated variables turn out to be the most important, this result will signal the relevance of that group of predictors. Indeed, if we were to remove collinearities a priori we would not be able to identify recurrent important variables (this was tested by eliminating highly correlated variables and retaining only one amongst them). Predictors are primarily selected and ranked using the standard Mean Absolute Error (MAE). Root-Mean-Square Difference (RMSD) and linear correlation were also computed, and results are essentially the same as for MAE.

### Variable ranking

This procedure consists in the ordering of the complete set of all the 121 variables, and it is based on a forward step-wise methodology which retains a variable at the time, starting with no variables and recursively adding the variable which yield the largest MAE reduction of the GCP-PC [25]. A k-fold (with k equal to 5) cross validation procedure is performed to estimate the MAE prediction statistic. The k-fold validation is commonly used to test the performance of a prediction model, particularly when there are not independent data for validation [25]. Essentially, a sample is divided into k equally populated randomly selected sub-samples. In turn, (k-1) sub-sample(s) is/are used for the training and the remaining sub-sample is used for the prediction. The most typical values of k are 5 or 10.

Four samples are considered:

1. All grid points (namely most of the world);
2. Grid points in the top (or upper) third (or tercile) of the GCP-PC distribution;
3. Grid points in the middle tercile of the GCP-PC distribution;
4. Grid points in the bottom (or lower) tercile of the GCP-PC distribution.

As well as identifying the explanatory variables for regions with different productivity (with samples 2, 3 and 4), these samples also allow to reduce the potential geographical correlation of GCP, in addition to the use of random sampling applied to the k-fold validation. Moreover, we also use the predictive power of the RF model to reconstruct the GCP-PC global map given the retained top geography and climate predictors, utilising the *k*-fold approach.

## Variable ranking and GCP per capita prediction

### Global GCP per capita

We start by presenting the comparison of the performance of the three statistical models tested: RF, GB and ML. Specifically, we run a comparison between RF and GB keeping all the 121 variables, using the k-fold validation approach, as well as with the top 10 predictors. The ML model is tested with the 10 selected predictors to avoid collinearities issues. As shown in Table 3, the RF’s error is less than half that of GB in terms of the normalised Mean Absolute Error (nMAE, i.e. MAE divided by the standard deviation of the log10 GCP-PC distribution). Similarly to the case with all variables, the RF performance for the top 10 predictors is more than twice as better than that of GB. While the first comparison, with all the variables, is more representative as the selection of 10 predictors has been made using the RF model, even the nMAE for the RF’s 10 predictors is distinctly lower than the GB’s 121 predictors. ML performs considerably worse than either RF and GB: the nMAE for ML is almost four times larger than that of RF. Tests with ML have been conducted also with 50 or 100 variables, in bootstrap mode, but the nMAE remains relatively high and equal to about 0.45 and 0.40, respectively. As an additional check, the performance of the RF model is also tested using the out-of-bag (OOB) prediction statistics (this is computed using withheld data within the sample used by the RF trees, and a useful benchmark).

**Fig 3:**
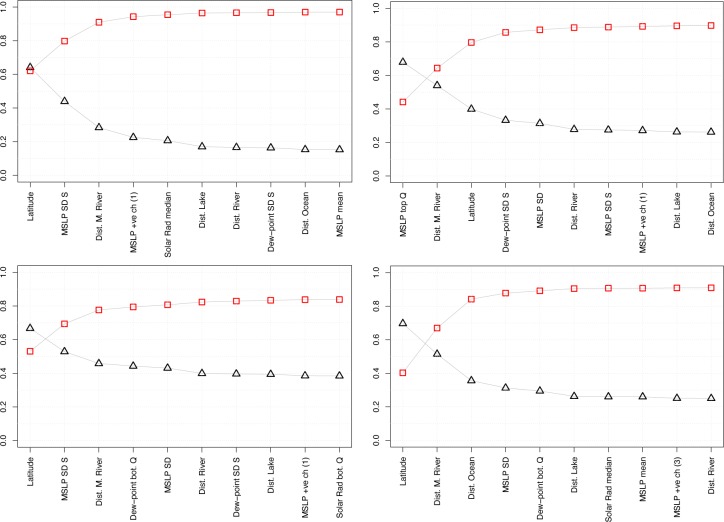
Summary statistics of GCP-PC prediction. Statistics of log_10_ GCP-PC prediction using 5-fold cross validation, adding one predictor at the time (forward step-wise approach) in the order given and up to 10 predictors. For instance, in the top left panel ‘MSLP SD S’ means that ‘MSLP SD S’ and ‘Latitude’ are used together (cf. also Table 4). Top left: all grid points. Top right: top tercile of GCP-PC. Bottom left: middle tercile of GCP-PC. Bottom right: bottom tercile of GCP-PC. Metrics used are: linear correlation (red squares) and normalised Mean Absolute Error (nMAE, black triangles). nMAE is equal to 0.56 for all grid points, 0.17 for the top and mid terciles, 0.26 for the bottom tercile.

The ranked top 10 variables are shown in Table 4 and in Fig 3. The latter shows how the nMAE, and the correlation, levels off after six-seven predictors, even if some small error reductions are seen with the subsequent predictors. Thus, while we retain the top 10 variables (as a round number) to display the asymptotic behaviour of the error, the focus should mainly be on the top five-six variables.

**Table 4:** Top 10 predictors and (*) their one-to-one linear correlation coefficient with GCP-PC for the cases of all-grid-point, top tercile, middle tercile, and bottom tercile. Green cells indicate predictors in common with the four cases, light blue in common with three cases and purple in common with two cases. Highlighted in salmon are the largest one-to-one correlation values for each of the four cases.

	All grid points		Top tercile		Middle tercile		Bottom tercile	
Rank	Predictor	Corr.*	Predictor	Corr.*	Predictor	Corr.*	Predictor	Corr.*
1	Latitude	0.35	MSLP top Q	0.05	Latitude	0.24	Latitude	0.30
2	MSLP SD S	-0.05	Dist. M. River	0.06	MSLP SD S	0.08	Dist. M. River	-0.13
3	Dist. M. River	-0.07	Latitude	0.06	Dist. M. River	-0.09	Dist. Ocean	-0.03
4	MSLP +ve ch (1)	0.42	Dew Point SD S	0.00	Dew Point bot. Q	-0.36	MSLP SD	0.33
5	Solar Rad median	0.45	MSLP SD	0.07	MSLP SD	0.35	Dew Point bot. Q	-0.23
6	Dist. Lake	0.00	Dist. River	-0.03	Dist. River	-0.16	Dist. Lake	0.20
7	Dist. River	0.06	MSLP SD S	-0.22	Dew Point SD S	0.29	Solar Rad median	-0.18
8	Dew Point SD S	0.23	MSLP +ve ch (1)	-0.23	Dist. Lake	-0.28	MSLP mean	0.18
9	Dist. Ocean	-0.08	Dist. Lake	-0.05	MSLP +ve ch (1)	0.33	MSLP +ve ch (3)	0.32
10	MSLP mean	0.26	Dist. Ocean	0.06	Solar Rad bot. Q	-0.36	Dist. River	0.17

**Fig 4:**
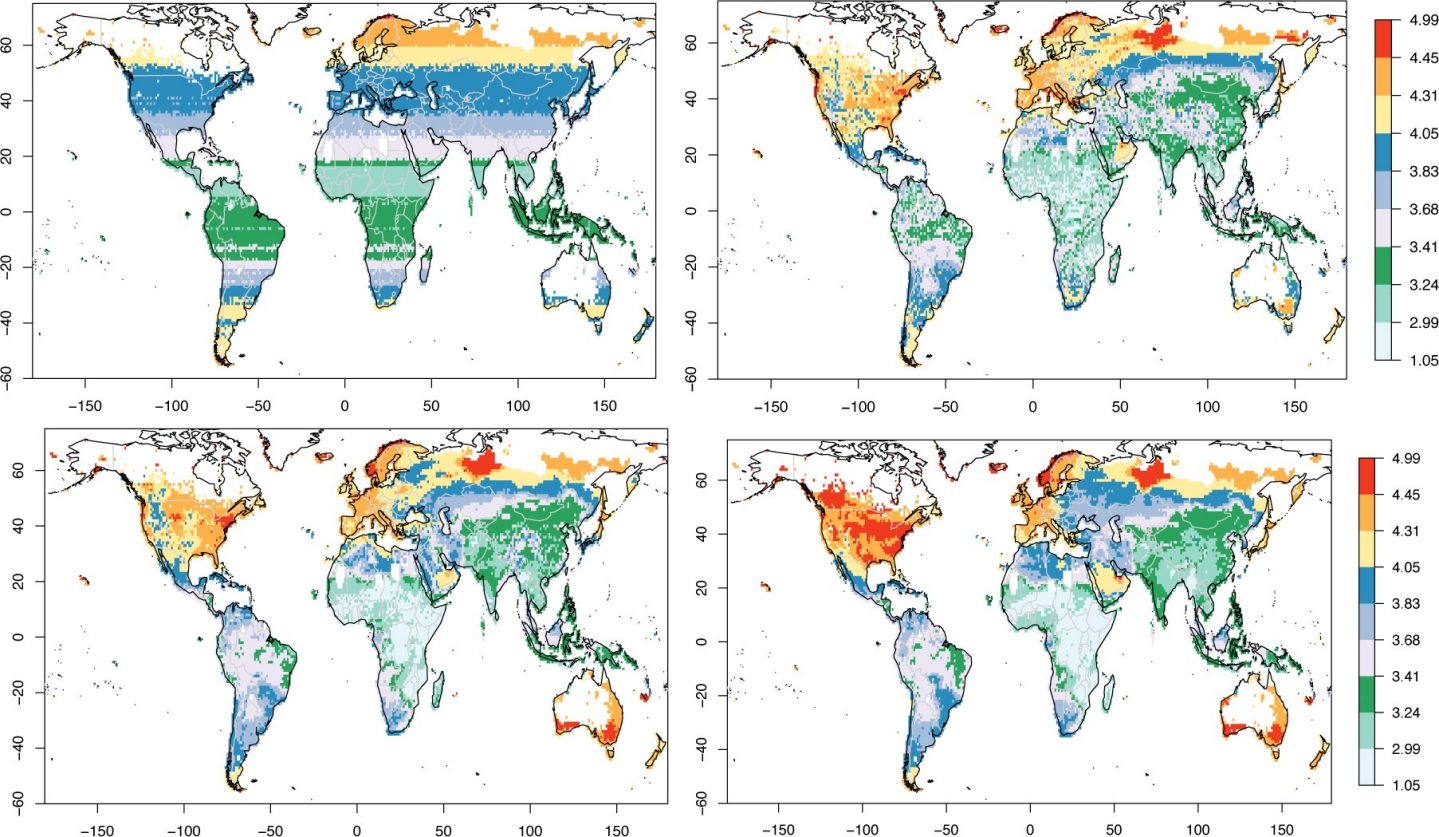
Maps of GCP-PC prediction. GCP-PC prediction (log_10_ of k USD/person) for the global case using the top predictors (see Table 4). Top left: top predictor. Top right: top two predictors. Bottom left: top three predictors. Bottom right: top six predictors. Compare with the actual GCP-PC in Fig 1.

Our analysis confirms that *latitude* is the dominant predictor for GCP-PC. However, it is also important to note that the nMAE with latitude only is around 0.65, hence relatively large, compared to the asymptotic value of ca. 0.15, obtained when at least six predictors are used. The limitations of using latitude-only are also apparent from the geographical reconstruction map (see Fig 4, top left). It is also worth noting that the mean air temperature, which is sometimes used as the main geographical variable instead of latitude in economic studies (e.g. [9]), in our data has a large, but not excessively so, linear correlation with latitude, namely 0.65. Somewhat unexpectedly, however, the second most important variable is a meteorological variable, and specifically MSLP SD S, namely the month-to-month variations of mean sea level pressure (or just air pressure). This variable reduces the nMAE to 0.44, when added to latitude. Not only is this meteorological variable more important than other geography variables, critically it is also not one that is normally considered in the literature (typically these are mean temperature or precipitation). And while air pressure technically encapsulates information about temperature, it is influenced by several other meteorological variables, like humidity. Importantly, this is not the standard *average* variable but a measure of variability, in this case an indication of seasonal (or intra-annual) variations. Also, while it would be inaccurate to identify MSLP SD S directly with specific meteorological phenomena such as the passage of a storm, this statistical measure contains the signature of storms. Therefore, this result seems to corroborate the more geographically and temporally limited finding of [2]. Additional evidence is discussed in section ‘*Patterns of top climate variables and potential physical link with GCP-PC*’.

The third most important variable in this ‘all grid points’ case is a geography variable, the distance from a major river. This variable reduces the nMAE to 0.28. The implications of the proximity of a major river are evident from a transport, recreational, etc. view point, but it is also worth mentioning that a link exists between rivers and environmental conditions such as water availability or soil conditions, therefore with clear connections to meteorological variables such as precipitation, temperature and humidity. The fourth variable is another meteorological variable, namely the step-to-step variations of air pressure at 6-hour intervals that are greater than the set ‘gradient’ threshold (see Table 1) and is expressed as the percentage of times this threshold is exceeded. This variable reduces the nMAE to ca. 0.23. An increase in air pressure, as expressed by this variable, is normally associated with drier and generally fairer weather. As with MSLP SD S, we see here again that the ‘traditional’ mean temperature and precipitation, while indirectly influencing air pressure do not rank amongst the most important variables in our analysis. Instead, *variations*, rather than mean values, appears to be emerging as most critical factors for explaining the geographical distribution of GCP-PC.

The fifth variable is the median of solar radiation which reduces nMAE by a further 0.02. Although this variable is less prominent than the variations in air pressure in our results, it is amongst the meteorological factors which has been found to affect mood or behaviour [26]. As with the third variable, the sixth (distance to lakes), the seventh (distance to a river) and the ninth (distance to ocean) most important variables are of geographical nature, as opposed to (purely) meteorological. Instead, the eight and tenth variables, in the top ten list, are again of meteorological nature. As noted above, the marginal reduction of the nMAE beyond the sixth predictor is minimal–nMAE drops by 0.02 from the sixth to the tenth predictor. Note also that air pressure enters the top ten list in three different ways: month-to-month variations, positive one-step ‘gradient’ and its mean. This is a clear indication that air pressure is a critical variable for explaining the GCP-PC.

**Fig 5:**
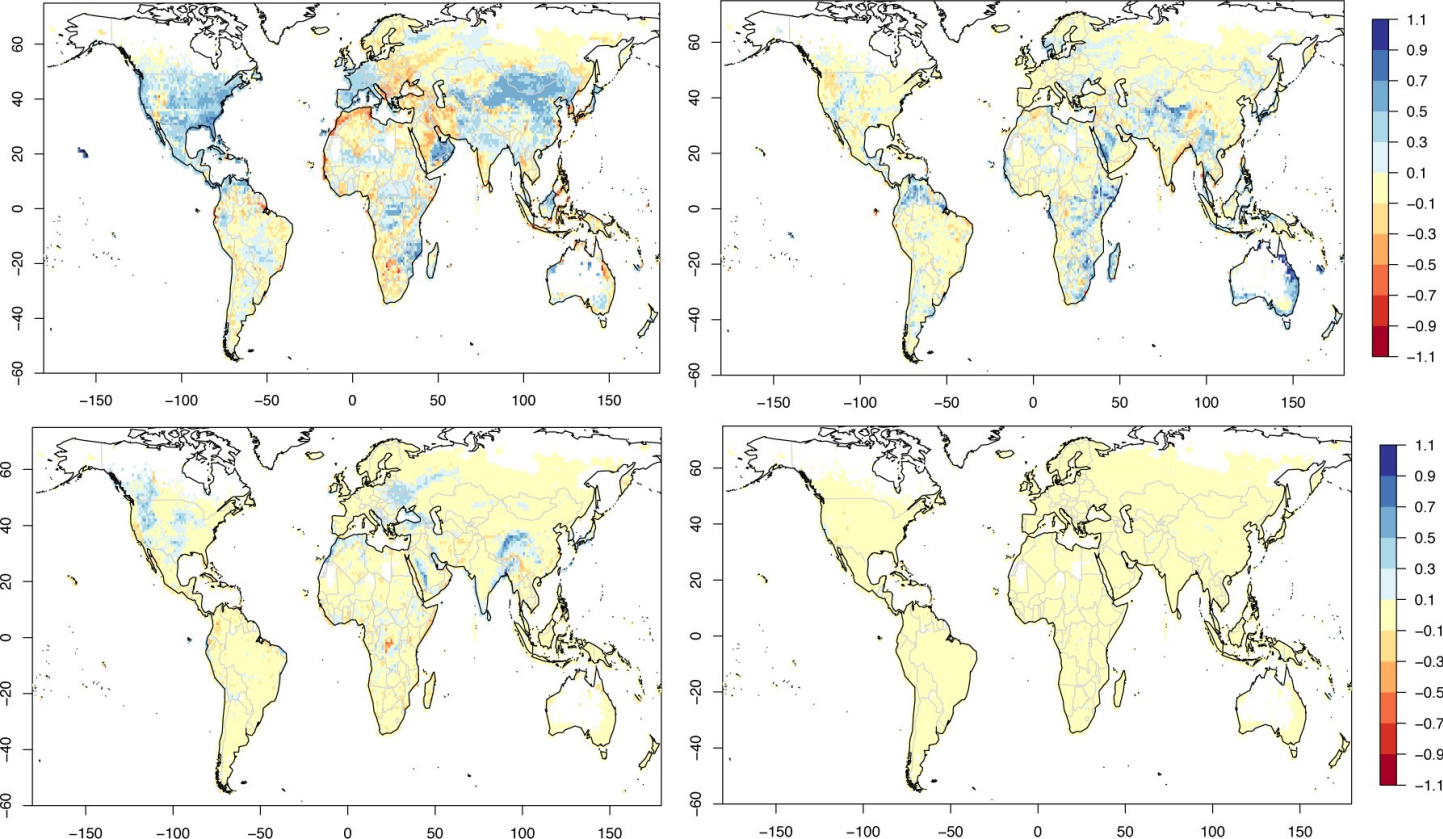
Maps of GCP-PC prediction departures. Changes in prediction expressed as the difference between the individual absolute departures from the actual GCP-PC values for step ‘n-1’ minus step ‘n’ for the global case. A positive (negative) value means that the additional predictor is improving (deteriorating) the prediction. Top left: Change from step 1 to 2. Top right: Change from step 2 to 3. Bottom left: Change from step 3 to 4. Bottom right: Change from step 6 to 7.

The maps with the geographical reconstruction of GCP-PC are shown in Fig 4. The top left GCP-PC map only uses latitude (the top variable) as its predictor. This is apparent from the zonal stripes, and with GCP-PC generally increasing with the absolute value of latitude. It is also evident that using latitude by itself, it is not possible to capture regional GCP-PC variations. These are introduced when the second predictor (MSLP SD S) is also considered. Now the regional fit is considerably improved, to the extent that areas in the Arabian peninsula, but also India, Central Africa and several other parts of the globe, now display a higher GCP-PC than other locations at the same latitude, and captured by the latitude predictor alone. This improvement is more clearly shown in Fig 5 (top left). Here the change in prediction from one step to the next is expressed as the difference between the individual absolute departures from the actual GCP-PC values for prediction ‘n-1’ minus prediction ‘n’ and calculated as follows:

ΔGCP‐PC=|Prediction(n‐1)–GCP‐PC|–|Prediction(n)–GCP‐PC| (2)

A positive (negative) value means that the additional predictor at step ‘n’ is improving (deteriorating) the prediction obtained at the previous step ‘n-1’.

Although the introduction of the second predictor generally improves the fit, there are also areas where the fit deteriorates, such as in Eastern Europe, North West Africa, Southern Africa, South Western USA, and North Eastern Australia. Some of these, particularly the latter one, are rectified when the third predictor (distance to a major river) is used (bottom left in Fig 4 and top right in Fig 5). Others, specifically Eastern Europe, North West Africa and South Western USA, then improve with the fourth predictor (MSLP positive step change) as seen in the bottom left panel of Fig 5. The bottom right panel in Fig 4 shows the geographical fit with the top six predictors, after which the error decreases only marginally (cf. Fig 3). This is confirmed by the bottom right panel of Fig 5 which shows that the change in GCP-PC prediction when the top seven variables are used (compared to the top six) is close to zero in most areas of the globe.

**Fig 6:**
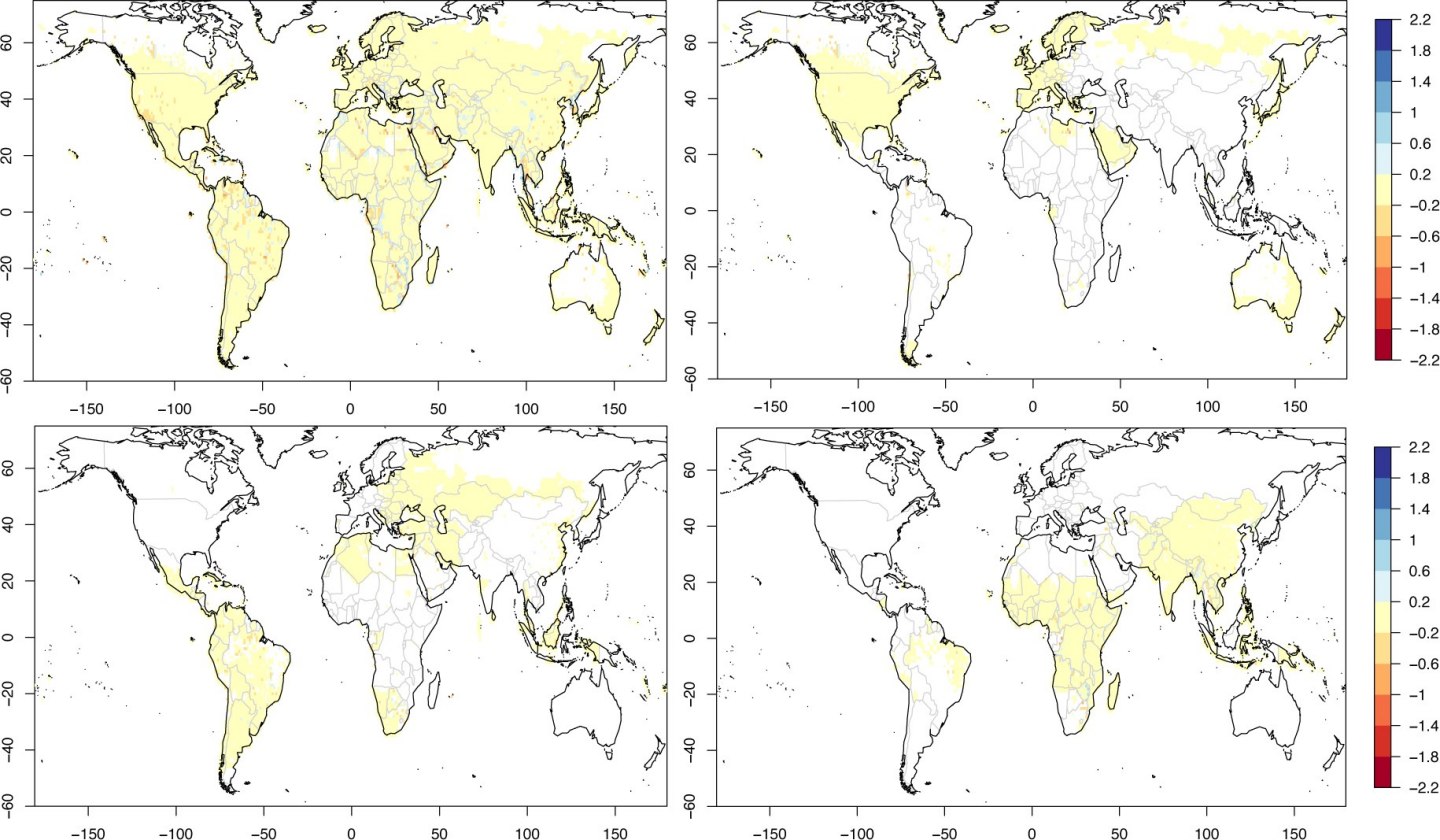
Difference between predicted and observed GCP-PC. Difference between predicted (using the top 10 predictors) and observed GCP-PC, namely (*f*–GCP-PC) in Eq (1). Top left: all grid points. Top right: top tercile of GCP-PC. Bottom left: middle tercile of GCP-PC. Bottom right: bottom tercile of GCP-PC.

While some noticeable differences between the predicted (using the top 10 predictors) and the measured GCP-PC can be seen (Fig 6 top left), these appear to be due, for the most part, to non-systematic errors. Possible exceptions, with consistent discrepancies either over a single country or a relatively large area, are: Gabon (central West Africa), South-West USA (parts of California and Arizona), and a few Balkan countries (Albania, Montenegro, and Bosnia and Herzegovina). Specifically, the latter area displays a negative bias, with the geography-climate prediction underestimating GCP-PC, and vice-versa in the former two cases. According to Eq (1) these discrepancies may indicate that the combined contribution of institutions and national resources (term *δ*) is not negligible: this term would be positive in the case of the Balkan countries above, with institutions and/or national resources therefore boosting economic productivity compared to the geography and climate baseline, and negative in the other two areas. However, as we will show later, these discrepancies are not present when smaller sections of the GCP-PC data (i.e. split into terciles) are modelled, which may imply that the results for the all-grid case exhibit a minor shortcoming in the RF statistical modelling with a very large amount of data being fit (nearly 13,000 grid points) and/or in the GCP-PC data, particularly for countries where the reporting of economic productivity figures is less established.

### Top tercile of GCP per capita

Here we subsample the GCP dataset by retaining only the top tercile of GCP-PC. The RF error again levels off after six or seven variables. From an initial nMAE of 0.68 with one predictor, this reaches a value of 0.28 after six variables and only improves by about 0.02 with the addition of the following four predictors–the nMAE with the top ten predictors is 0.26 (Fig 3 and Table 3). Compared to the all-grid-point case, latitude is not the most important predictor for the top tercile; rather it now ranks third, after the top quartile of air pressure (MSLP top Q) and the distance from major rivers. The top predictor, MSLP top Q, indicates that high air pressure is a key factor in determining the GCP-PC for countries in the top tercile. Also, and as in the case of all-grid-point, variations in climatic variables, rather than their mean, are still very important: the month-to-month variations for the dew-point temperature (despite having temperature in its name, dew point temperature, rather confusingly, is not a temperature; while it also depends on temperature, it is closely associated with relative humidity) and the overall variations of air pressure (MSLP SD) rank in the top five most important variables. The nMAE is reduced to 0.31 after the fifth variable, MSLP SD. Next, in sixth position, distance to rivers is again an important predictor, with a further reduction of nMAE by ca. 0.04.

Table 4 directly compares the top ten predictors in both the all-grid-point and top tercile cases. It is evident the large overlap amongst them, with eight variables in common (see coloured boxes), and another variable related to air pressure in both cases (MSLP top Q and MSLP mean).

The RF model yields again a remarkable geographical prediction/reconstruction of the top tercile of GCP-PC (S3 Fig). While the top quartile of air pressure (top predictor) sufficiently reproduces the overall geographical pattern of GCP-PC, subsequent predictors clearly enhance the fit (S4 Fig). For instance, evident improvements over a few areas in Russia, Libya, the Arabian peninsula and northern/western Australia are seen with the inclusion of the distance from major rivers. Latitude and the month-to-month variations of dew-point temperature further noticeably improve the fit to the GCP-PC, particularly over Libya, southern USA and northern Mexico in the case of latitude, and Arabian peninsula and Spain in the case of dew-point temperature. Subsequent predictors only modify marginally the fit. When compared with the measured GCP-PC, there do not seem to be systematic biases in the predicted (using the top 10 predictors) GCP-PC (Fig 6 top right).

### Mid and bottom terciles of GCP per capita

To complement the analysis of the top tercile, we concisely discuss the results for the middle and bottom terciles of GCP-PC. In terms of overall statistics (Table 3), the middle terciles yields slightly worse results than the top tercile across most of the metrics, notably for the RF performance, which is however still considerably superior to GB, and ML. The bottom tercile, instead, yields very marginally better results than the top tercile. Further, in terms of actual predictors, Table 4 shows that there is a good level of agreement for all four cases, with four predictors out of ten in common when all four cases are taken together, with further four-five in common amongst three cases. When cases are taken in pairs the number of common predictors range between six and eight predictors. Further, even in the case of the middle and bottom terciles the RF model is capable of reproducing well the observed respective GCP-PC using the top six predictors (S5–S8 Figs). This assessment demonstrates that the statistical, and physical, link between GCP-PC and geography and climate variables is robust. As with the top tercile, there do not seem to be systematic biases in the predicted (using the top 10 predictors) GCP-PC (Fig 6 bottom panels), except perhaps for a couple of areas in the case of the bottom tercile: north-west Myanmar and around the border of Zimbabwe (Fig 6 bottom right), though these are likely due to GCP-PC data issues.

### Behaviour of top variables and their potential physical link with GCP-PC

We now take a closer look at the behaviour of the top predictors, particularly for the all-grid-point case, as a way to gain a better understanding of how such predictors affect GCP-PC, focussing especially on the climatic variables. Table 4 shows the linear correlation coefficient between the top ten predictors and the corresponding GCP-PC, for all-grid-point and the three terciles, respectively. It is interesting to note that individually none of the predictors has a very high linear correlation with GCP-PC, in any of the four cases. The highest correlation coefficients are with Solar Radiation median (0.45), MSLP positive step-one change (0.42), Dew Point bottom Q (0.36), Solar Radiation bottom Q (0.36) and latitude (0.35). The first two and latitude occur in the all-grid-point case, whereas the other two are for the middle tercile. It may appear odd that despite Solar Radiation median and MSLP positive step-one change have a higher linear correlation with GCP-PC than latitude in the all-grid-point case, it is the latter that ranks first in importance. This behaviour may be explained through the following assessment.

**Fig 7:**
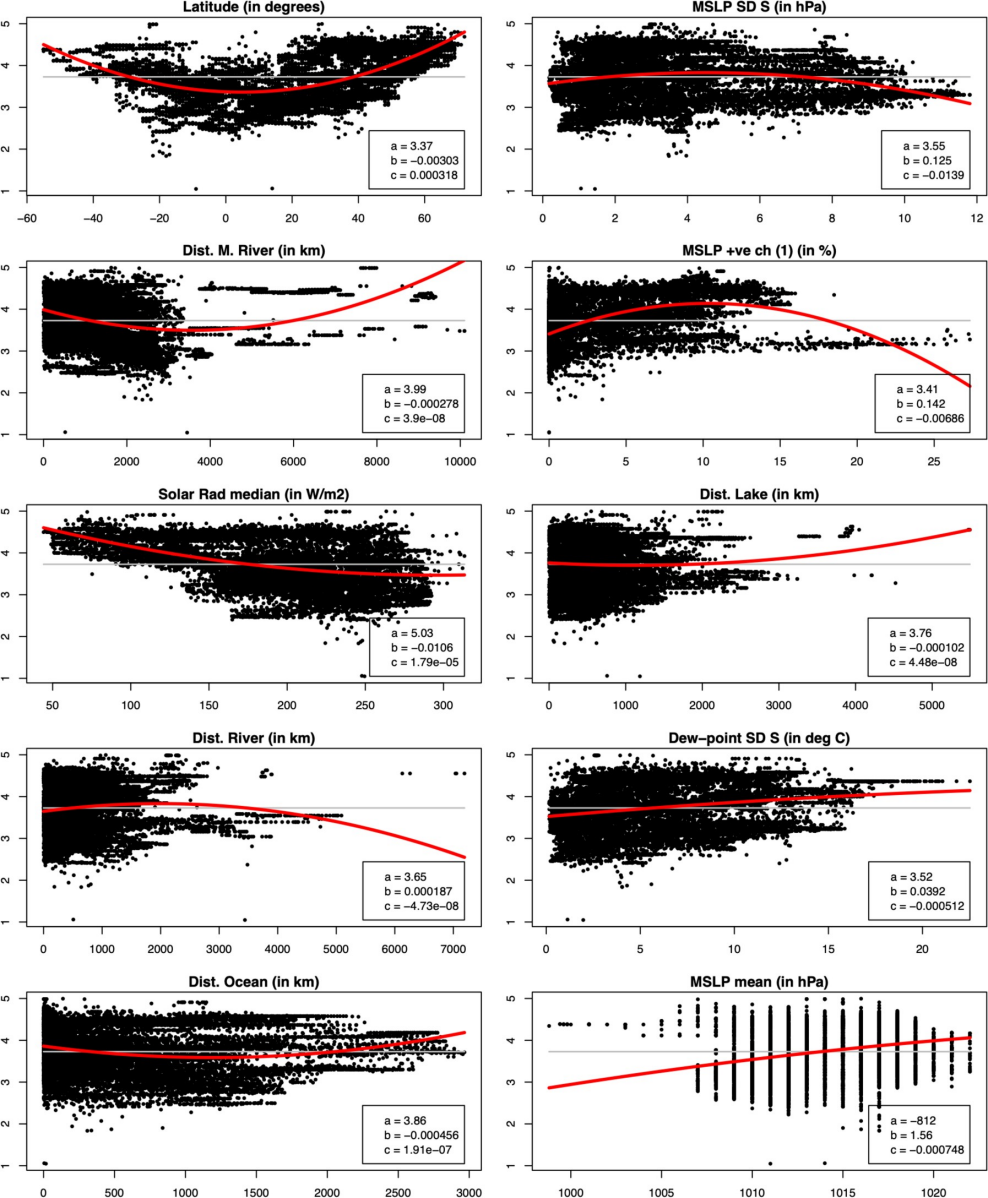
Scatter plots and quadratic fit of the top ten predictors with GCP-PC. Diagrams showing the distribution (scatter) of the top predictors (x-axis) for the all-grid-point case versus GCP-PC (y-axis). The red line is the quadratic fit, as given by the equation *y* = a + b *x* + c *x*^2^, with the corresponding parameters (a, b, c) indicated in each panel. As a reference, the grey line indicates the average GCP-PC (cf. Fig 2).

For illustrative purposes, we consider the quadratic fit of the top ten predictors for the all-grid-point case. While generally there is a wide scatter between each predictor and GCP-PC, it also apparent that the relationship between individual predictors and GCP-PC is not a linear one (Fig 7). Thus, whether the most appropriate line fit is quadratic or a more complex one, these plots give an indication as of why a non linear model such as RF is superior to a linear model. At the same time, it is important not to over-interpret the results of the quadratic fit. If we take for instance the third variable, distance from major rivers (second top row, left), it would appear that at distances larger than around 4000 km, namely the minimum of the quadratic curve, GCP-PC starts to increase again. This is essentially due to the lack of major rivers, as defined by [21], in countries such as Australia and New Zealand (see S8 Fig). On the other hand, the quadratic fit can provide some useful indications about the values of the variables that are linked to above or below GCP-PC average. For instance, in the case of latitude above average GCP-PC appears north of around 40°N and south of 30°S (see where red line crosses the grey line in Fig 7); in the case of MSLP SD S, higher than average GCP-PC appears for values between about 2 and 7 hPa; and for MSLP positive one-step change, above average GCP-PC appears for values between about 2.5 and 17.5%. This assessment is complemented by the regional geographical patterns of the top ten predictors presented in the supplementary information (S8 Fig).

Although the majority of the literature linking economic productivity-related factors such as physiology or labour allocation with weather and climate take only temperature, and sometimes precipitation, into account [27, 28, 29, 30], there is increasing evidence that meteorological variations affect human mood. It is long been known that mood, which can be categorised as concentration, cooperation, anxiety, depression, sleepiness, and other behaviours, can have significant impacts on human activities, and ultimately economic productivity [31].

Perhaps the most comprehensive reference for the effect of meteorological (variations) on mood is [26]. In this book, we read for instance that ‘*it is a more complex mix of weather variables including pressure drop*, *humidity and/or temperature that causes the greatest stress on the body and most likely leads to increased pain*’. Or that ‘*infrasound*, also *caused by fluctuations in atmospheric pressure*, *can easily penetrate buildings and affect their inhabitants*’ [26]. Physiology explanations of the effect of weather on human body date back to the 1930s with [32], where it is stated that when a weather ‘*front approaches and the atmospheric pressure begins to fall*, *the body responds by contracting blood vessels and reducing the amount of oxygen to the heart*, *brain*, *kidneys and other major organs*. *This causes blood pressure to rise*, *resulting in an overall ‘stimulation’ of the body*’. More specifically, [33] finds that pressure change, even more than pressure level, appears to be important in determining mood/behaviour. Similar findings are reported in [34, 35, 36]. Interestingly, [35] also found that effect of meteorological factors on mood is nearly the same whether people spend almost all of their time indoors (i.e., less than 30–45 min outside) or outdoors. Moreover, [36] found a correlation between the frequency of migraine headaches and the day-to-day difference in barometric pressure.

In the context of our results, and referring back to the identified ranges in the variables in Fig 7 and S8 Fig, their values appear to imply that higher economic productivity is linked with variations in meteorological variables that are moderate: too low values may not provide sufficient physiological and/or psychological stimulation [26], whereas too strong values, likely connected to adverse severe weather conditions, may lead to [national] incomes decline [37]. Clearly more research would be needed to firmly establish a link between meteorological variations and economic productivity–this is mainly limited by the samples currently available which do not allow a robust statistical characterization across geographies and meteorological conditions.

### Role of country fixed effects

While the focus of this work is on the role of exogenous geography and climate variables to explain GCP-PC, we want to try to distinguish whether the results obtained here are the effect of within-country variations or a world-wide (cross-country) variations. Such an assessment would assist with the assessment of the (relative) role of institutions on economic productivity.

A standard approach to testing the within- versus cross- country effects is the use of dummy variables, whereby each country in turn is given the value of one, while all the others are set to zero. These variables have the purpose of fixing the within country effects, even if setting a single (unit) value may not be a good approximation for some countries given their heterogenous institutions. This notwithstanding, together with the fact that we would need nearly two hundred dummy variables (corresponding to the number of countries considered here) which would make the interpretation of results extremely complex, the RF model does not lend itself to mixing categorical (dummy-variables) and continuous variables (geography and climate variables).

An alternative approach is to make use of one of the six institution-related (continuous) indicator listed in the Introduction [18]. As mentioned, these are only available as country averages, and therefore are not directly comparable to the climate and geography fine resolution data. Also, while they may represent within-country fixed effects, they do not distinguish between countries with similar values of the chosen institutional indicator. Even more critically, however, any such indicator is both endogenous and highly correlated with GCP-PC. As in [4], we consider here Rule of Law as the institutional indicator, by setting the same value for each grid point within its assigned country. Rule of Law has a correlation coefficient of 0.65 with GCP-PC. When Rule of Law is included as an additional predictor in our RF model, and ranked together with climate and geography variables, it yields (for the global case) an nMAE three time smaller (hence better) than for latitude alone (the top geography/climate predictor), and a correlation coefficient of 0.94 (compared to 0.62 for latitude alone). Stated otherwise, Rule of Law alone has the same explanatory power as the combined first 4–5 geography and climate predictors. This means that any additional geography/climate predictor, when added to Rule of Law, improves the fit with GCP-PC only marginally.

While such a test is useful in proving that an institutional indicator such as Rule of Law can explain GCP-PC to a high degree of precision within our modelling framework, this does not provide a definitive explanation of country level fixed effects–given the inability of Rule of Law to discriminate between countries with similar values, such as Germany and United Kingdom. More fundamentally, however, Rule of Law (as any other institutional indicator), being endogenous, is inextricably connected with GDP and it is therefore arduous to relate it to the exogenous climate and geography variables considered in this work.

## Conclusions and discussion

This work has investigated the role that exogenous factors represented by a wide range of climate and geography variables have on worldwide economic activity as measured by the per capita Gross Cell Product (GCP-PC) at a fine, 1° by 1°, geographical resolution. We considered two main cases: all global grid points and upper tercile of GCP-PC. We find that eight out of the top ten predictors are in common in these two cases. However, an interesting distinction is that latitude is the top predictor in the first case, but it is less important in the second case (it ranks third). We have also seen that month-to-month variations of meteorological variables, as well as their variations–particularly mean sea level pressure and dew point temperature–are the main climate predictors that explain economic activity worldwide. Interestingly less than ten variables, and usually six-seven variables explain around 80% of the variance in GCP-PC.

For completeness, the middle and bottom terciles of GCP-PC have also been modelled. The simulations and predictions for these two cases provide further evidence that most of the economic activity, as represented by the GCP-PC, can be explained through a (limited number of) geography and climate predictors, even if the accuracy of the results considerably decreases for individual terciles in terms of nMAE compared to the global case. Further, and crucially, the results for the three terciles, as well as for the whole distribution, indicate a close agreement amongst each other in terms of the most important explanatory geography and climate predictors, with six variables, out of ten, in common when all four cases together are considered. This also demonstrates the robustness of the non-linear machine learning modelling framework adopted here.

While the role of institutions (and natural resources) has been marginally considered in this study, the fact that the global GCP-PC can be reproduced with a high level of accuracy by using a limited and recurring set of geography and meteorology variables, indicates that these are critical factors in explaining fine location-specific economic activities. Aside from the standard latitude indicator, it remains to be established exactly what the physical links between the identified most important explanatory variables, such as variations in air pressure, and economic productivity are: these links could be the object of a future study. Such a study would draw on the growing bio-meteorological, physiological and psychological literature that, as presented in this paper, relates meteorological variables and its variations, including of air pressure and humidity, to human mood and behaviour, and which in turn could affect economic productivity.

Our results may have other important implications such as the fact that the relationship between climate and economic activity in the recent past could provide an indication of what the climate conditions were in the distant past in relation to known economically active regions of the world (e.g. the once prosperous Mesopotamia). Conversely, knowing how the climate is projected to vary in the second half of this (XXI) century can give an indication of the possible future economic activity in various parts of the world. Another application could be the consideration of relevant geography and climate conditions to informing where new economic activities could be established to enhance favourable economic outcomes.

## Supporting information

S1 Fig Map of gross cell product. GCP in log10(k USD); this is also referred to as Gross Cell Product. Note the different scale than the one used for GCP-PC (e.g. in Fig 1). Source G-Econ [18].
(PDF)

S2 Fig Maps of GCP-PC prediction for top tercile. As in Fig 4 but for the top tercile of the GCP-PC distribution. Compare with the actual GCP-PC in Fig 1.
(PDF)

S3 Fig Maps of GCP-PC prediction departures for top tercile. As in Fig 5 but for the top tercile of the GCP-PC distribution.
(PDF)

S4 Fig Maps of GCP-PC prediction for middle tercile. As in Fig 4 but for the middle tercile of the GCP-PC distribution. Compare with the actual GCP-PC in Fig 1.
(PDF)

S5 Fig Maps of GCP-PC prediction departures for middle tercile. As in Fig 5 but for the middle tercile of the GCP-PC distribution.
(PDF)

S6 Fig Maps of GCP-PC prediction for bottom tercile. As in Fig 4 but for the bottom tercile of the GCP-PC distribution. Compare with the actual GCP-PC in Fig 1.
(PDF)

S7 Fig Maps of GCP-PC prediction departures for bottom tercile. As in Fig 5 but for the bottom tercile of the GCP-PC distribution.
(PDF)

S8 Fig Maps of main climatic and geographic predictors. Geographical features of the six main climatic (meteorological) and geography predictors: latitude (top left, in°), MSLP standard deviation seasonal (top right, in hPa), distance from major rivers (middle left, in km), MSLP positive one-step (6-hour) change (middle right, in %), solar radiation median (bottom left, in W m^-2^), distance from lakes (bottom right, in km). There is no marked correlation between these fields and GCP-PC, with the highest linear correlation being with the median of solar radiation (0.45, see also Table 4).
(PDF)
