# Supplementary material for: Effect of climate and geography on worldwide fine resolution economic activity
Source: PLoS One. 2020 Mar 2;15(3):e0229243. doi: 10.1371/journal.pone.0229243 (PMC7051056; doi:10.1371/journal.pone.0229243)
Supplement: S1 Fig — GCP in log10(k USD); this is also referred to as Gross Cell Product. Note the different scale than the one used for GCP-PC (e.g. in Fig 1). Source G-Econ [18]. (PDF) [file pone.0229243.s001.pdf]

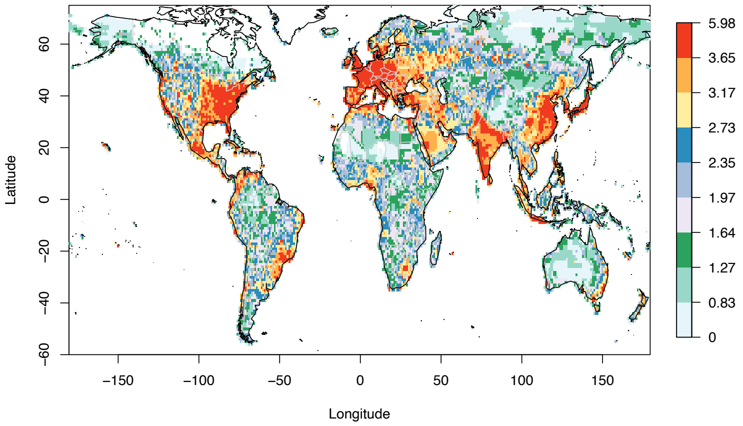

S1 Fig. Map of Gross Cell Product. GCP in $\log_{10}$(k USD); this is also referred to as Gross Cell Product. Note the different scale than the one used for GCP-PC (e.g. in Fig 1). Source G-Econ [18].
