# Supplementary material for: Effect of climate and geography on worldwide fine resolution economic activity
Source: PLoS One. 2020 Mar 2;15(3):e0229243. doi: 10.1371/journal.pone.0229243 (PMC7051056; doi:10.1371/journal.pone.0229243)
Supplement: S3 Fig — As in Fig 5 but for the top tercile of the GCP-PC distribution. (PDF) [file pone.0229243.s003.pdf]

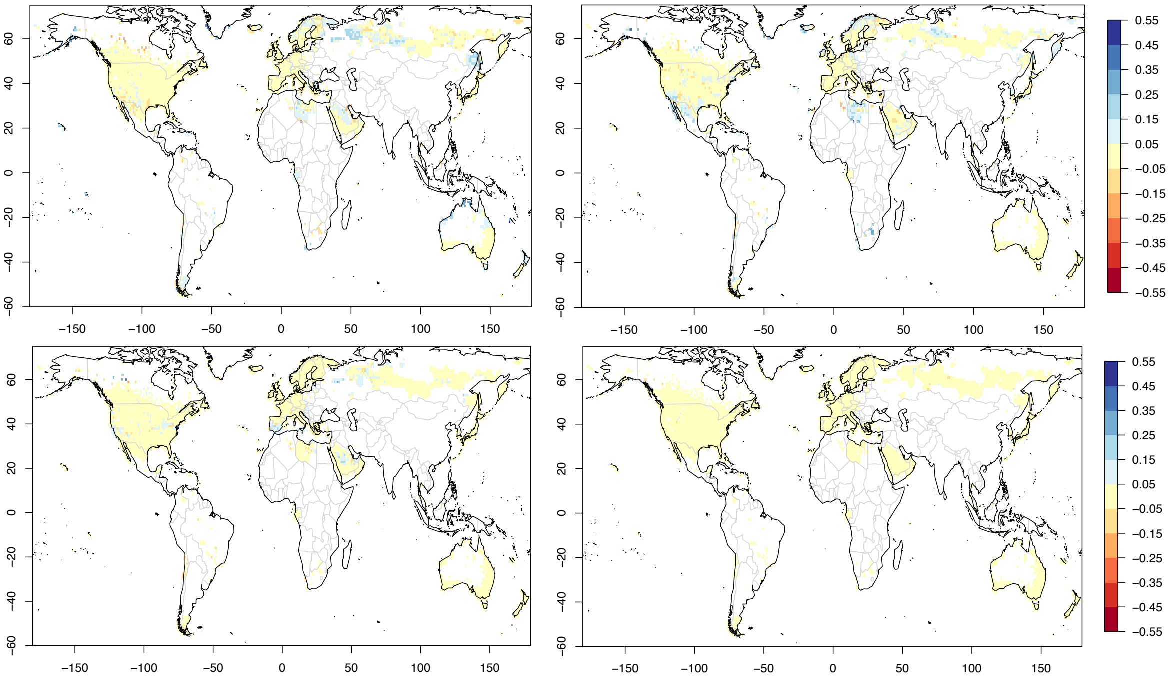

S3 Fig. Maps of GCP-PC prediction departures for top tercile. As in Fig 5 but for the top tercile of the GCP-PC distribution.
