# Supplementary material for: Effect of climate and geography on worldwide fine resolution economic activity
Source: PLoS One. 2020 Mar 2;15(3):e0229243. doi: 10.1371/journal.pone.0229243 (PMC7051056; doi:10.1371/journal.pone.0229243)
Supplement: S6 Fig — As in Fig 4 but for the bottom tercile of the GCP-PC distribution. Compare with the actual GCP-PC in Fig 1. (PDF) [file pone.0229243.s006.pdf]

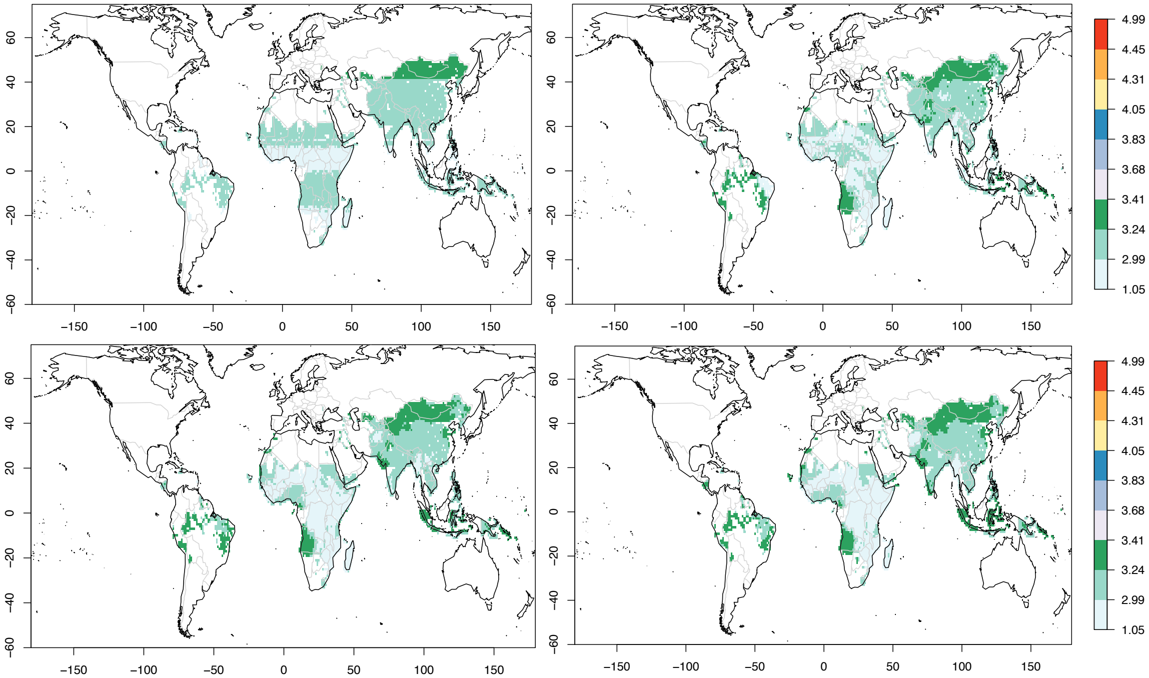

S6 Fig. Maps of GCP-PC prediction for bottom tercile. As in Fig 4 but for the bottom tercile of the GCP-PC distribution. Compare with the actual GCP-PC in Fig 1.
