# Supplementary material for: Effect of climate and geography on worldwide fine resolution economic activity
Source: PLoS One. 2020 Mar 2;15(3):e0229243. doi: 10.1371/journal.pone.0229243 (PMC7051056; doi:10.1371/journal.pone.0229243)
Supplement: S8 Fig — Geographical features of the six main climatic (meteorological) and geography predictors: latitude (top left, in°), MSLP standard deviation seasonal (top right, in hPa), distance from major rivers (middle left, in km), MSLP positive one-step (6-hour) change (middle right, in %), solar radiation median (bottom left, in W m-2), distance from lakes (bottom right, in km). There is no marked correlation between these fields and GCP-PC, with the highest linear correlation being with the median of solar radiation (0.45, see also Table 4). (PDF) [file pone.0229243.s008.pdf]

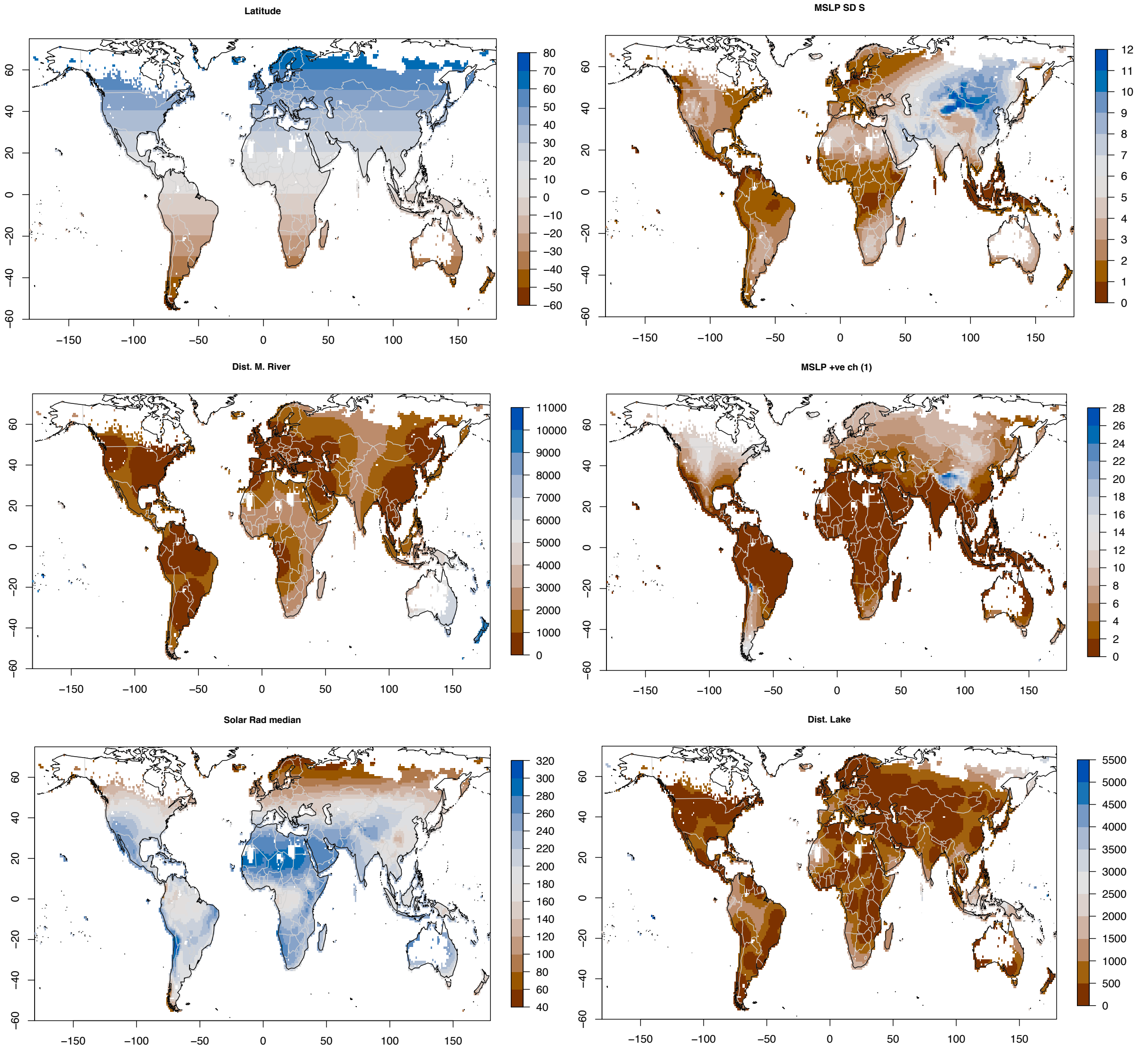

S8 Fig. Maps of main climatic and geographic predictors. Geographical features of the six main climatic (meteorological) and geography predictors: latitude (top left, in °), MSLP standard deviation seasonal (top right, in hPa), distance from major rivers (middle left, in km), MSLP positive one-step (6-hour) change (middle right, in %), solar radiation median (bottom left, in W m^{-2}), distance from lakes (bottom right, in km). There is no marked correlation between these fields and GCP-PC, with the highest linear correlation being with the median of solar radiation (0.45, see also Table 4).
